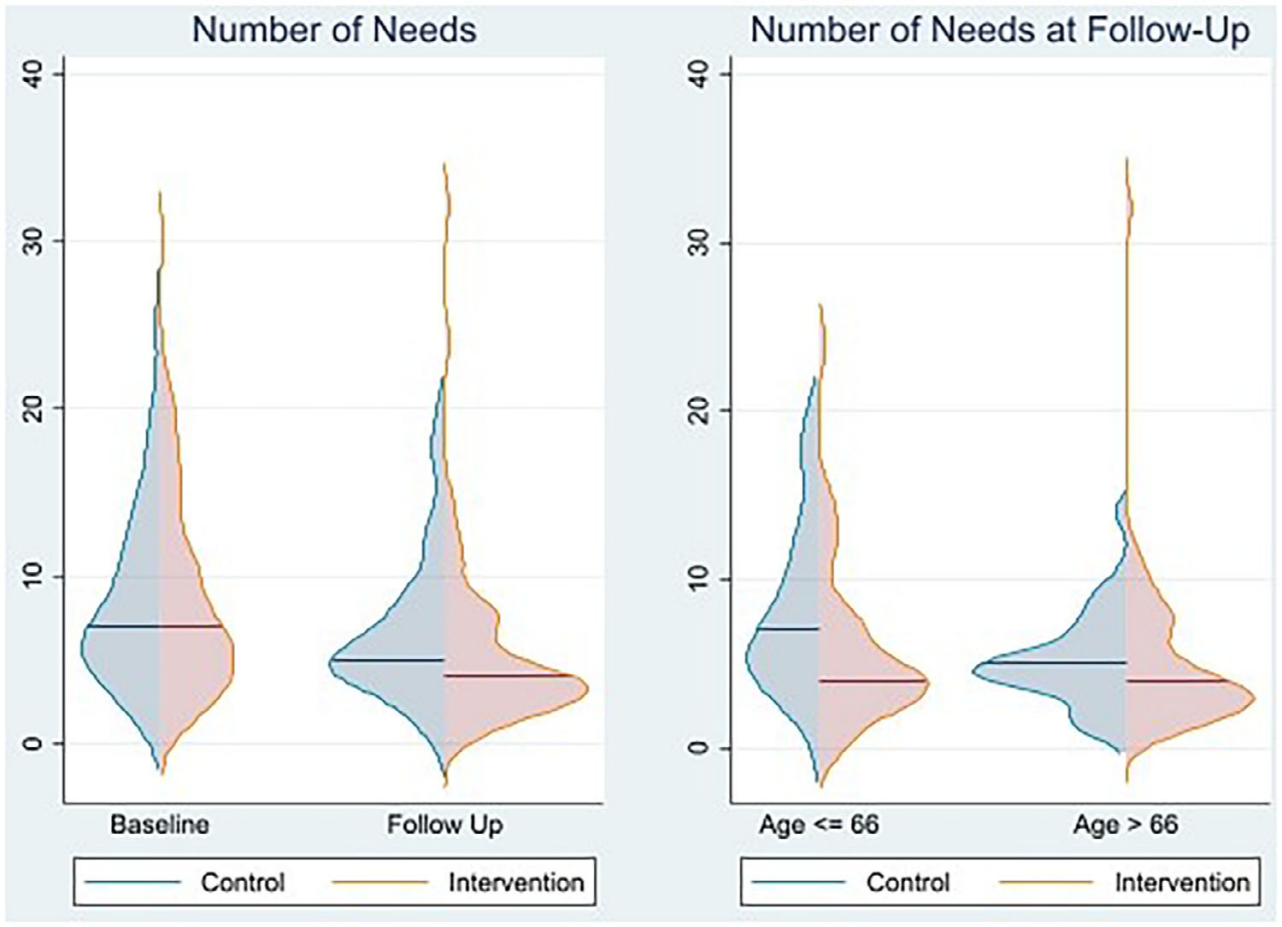# Enhancing Dementia Care: A Digitally Supported Care Management Program for Informal Caregivers ‐ A Cluster‐Randomized Controlled Trial

**DOI:** 10.1002/alz.088831

**Published:** 2025-01-09

**Authors:** Melanie Boekholt, Olga A. Klein, Iris Blotenberg, Stefan Teipel, Bernhard Michalowsky, Ingo Kilimann, Wolfgang Hoffmann, Jochen René Thyrian

**Affiliations:** ^1^ Deutsches Zentrum für Neurodegenerative Erkrankungen e. V. (DZNE), site Rostock / Greifswald, Greifswald Germany; ^2^ Deutsches Zentrum für Neurodegenerative Erkrankungen e. V. (DZNE), Rostock Germany; ^3^ Deutsches Zentrum für Neurodegenerative Erkrankungen e. V. (DZNE) Rostock/Greifswald, Rostock Germany; ^4^ Klinik und Poliklinik für Psychiatrie und Psychotherapie, University of Rostock, Rostock Germany; ^5^ Department of Psychosomatic Medicine, Rostock University Medical Center, Rostock Germany; ^6^ German Center for Neurodegenerative Diseases e.V. (DZNE), Site Rostock/Greifswald, Greifswald Germany; ^7^ Institut for Community Medicine, University Medicine, University of Greifswald, Greifswald Germany; ^8^ Deutsches Zentrum für Neurodegenerative Erkrankungen e.V. (DZNE) and Institute for Community Medicine / University of Greifswald, Greifswald Germany; ^9^ Institute for Community Medicine / University of Greifswald, Greifswald Germany

## Abstract

**Background:**

Approximately 1.8 million individuals in Germany live with dementia, imposing a substantial burden on family caregivers who provide most care and often experience health issues, social isolation, and diminished quality of life. Recognizing and addressing the diverse needs of these caregivers is vital for their well‐being and the stability of care arrangements.

**Method:**

This study employed a longitudinal, cluster‐randomized controlled trial in Mecklenburg‐Western Pomerania, Germany, with 192 informal caregivers of people living with dementia. Participants were randomly assigned to either the control (n = 96) or intervention (n = 96) group. The intervention involved a digitally supported care management program delivered by trained care managers, specifically tailored to address the unmet needs of caregivers.

**Result:**

Primary outcomes after 6 months included the number of unmet needs and the health‐related quality of life of caregivers. Secondary outcomes comprised caregiver burden and social network/support. While the intervention did not significantly reduce overall unmet needs in multivariate models, it demonstrated a notable effect in the subgroup of younger family caregivers. No statistically significant impacts were observed on other outcomes.

**Conclusion:**

Individualized care management, as delivered through this program, effectively addresses the specific support needs of informal caregivers. The study emphasizes the importance of tailoring interventions to sustain preferred caregiving arrangements and underscores the necessity of investigating nuanced needs for effective support. Although the program did not show a universal reduction in unmet needs, its positive impact on younger caregivers suggests potential benefits for specific demographic groups.